# Differential Regulation of Methylation-Regulating Enzymes by Senescent Stromal Cells Drives Colorectal Cancer Cell Response to DNA-Demethylating Epi-Drugs

**DOI:** 10.1155/2018/6013728

**Published:** 2018-08-12

**Authors:** Khushboo Agrawal, Viswanath Das, Natálie Táborská, Ján Gurský, Petr Džubák, Marián Hajdúch

**Affiliations:** ^1^Institute of Molecular and Translational Medicine, Faculty of Medicine and Dentistry, Palacky University, Hněvotínská 5, 77900 Olomouc, Czech Republic; ^2^Cancer Research Czech Republic, Hněvotínská 5, 77900 Olomouc, Czech Republic

## Abstract

The advanced-stage colon cancer spreads from primary tumor site to distant organs where the colon-unassociated stromal population provides a favorable niche for the growth of tumor cells. The heterocellular interactions between colon cancer cells and colon-unassociated fibroblasts at distant metastatic sites are important, yet these cell-cell interactions for therapeutic strategies for metastatic colon cancer remain underestimated. Recent studies have shown the therapeutic potential of DNA-demethylating epi-drugs 5-azacytidine (AZA) and 5-aza-2′-deoxycytidine (DAC) for the treatment of solid tumors. While the effects of these epi-drugs alone or in combination with other anticancer therapies are well described, the influence of stromal cells and their secretome on cancer cell response to these agents remain elusive. In this study, we determined the effect of normal and senescent colon-unassociated fibroblasts and their conditioned medium on colorectal cancer (CRC) cell response to AZA and DAC using a cell-based DNA demethylation reporter system. Our data show that fibroblasts accelerate cell proliferation and differentially regulate the expression of DNA methylation-regulating enzymes, enhancing DAC-induced demethylation in CRC cells. In contrast, the conditioned medium from senescent fibroblasts that upregulated NF-*κ*B activity altered deoxycytidine kinase levels in drug-untreated CRC cells and abrogated DAC effect on degradation of DNA methyltransferase 1. Similar to 2D cultures, senescent fibroblasts increased DNA demethylation of CRC cells in coculture spheroids, in addition to increasing the stemness of CRC cells. This study presents the first evidence of the effect of normal and senescent stromal cells and their conditioned medium on DNA demethylation by DAC. The data show an increased activity of DAC in high stromal cell cocultures and suggest the potential of the tumor-stroma ratio in predicting the outcome of DNA-demethylating epigenetic cancer therapy.

## 1. Introduction

Colorectal cancer (CRC) is one of the most common cancers with heterogeneous treatment outcomes [1, 2], and growing evidence indicates the key role of the stroma in CRC invasion, metastasis, and response to chemo- and radiotherapy [3–5]. An image-based quantitative study conducted in CRC patient samples suggests the abundance of cancer-associated fibroblasts in tumor stroma as an indicator of disease recurrence after curative CRC surgery [6]. In poor-prognosis CRC subtypes that are characterized by stemness and/or epithelial-to-mesenchymal transition (EMT), elevated expression of mesenchymal genes is mainly contributed by tumor-associated stroma [7]. High Wnt signaling activity in tumor cells that are located close to stromal myofibroblasts further indicates that stemness of colon cancer cells is partly regulated by the tumor microenvironment [8].

The cellular heterogeneity in the tumor microenvironment plays a key role in tumor progression, invasion, metastasis, and the outcome of anticancer therapy [9]. While the tumor stroma is not malignant per se, stromal cells acquire abnormal phenotype and support the growth and progression of cancer [9, 10]. Importantly, the role of senescent stromal cells in the tumor microenvironment is coming into light due to their ability to drive the unrestrained growth of tumors, which cause a differential response of cancer cells to anticancer drugs [11, 12]. Senescence is one of the normal cellular events triggered in cancer cells following genotoxic stress, such as radiotherapy and chemotherapy [13]. However, therapy-induced bystander senescence in other noncancerous cell types of the tumor microenvironment has been suggested to result in cancer relapse and aggravate the side effects of chemotherapy [12, 14, 15]. Therefore, there is a growing interest to understand how senescent stromal cells alter the response of tumor cells to different classes of anticancer drugs [16].

DNA methyltransferase inhibitors (DNMTIs), such as 5-azacytidine (AZA) and 5-aza-2′-deoxycytidine (DAC), have shown promising activity as priming agents in the treatment of solid tumors in early clinical trials [17–20]. DNMTIs have been reported to work synergistically in combination with various other anticancer therapies [21–25] and radiotherapy [26, 27]. Although the effects of DNMTIs alone or in combination with radiotherapy are well reported, it is not known how the senescent and/or normal stromal cells of the tumor microenvironment influence the response of cancer cells to DNA-demethylating drugs. Besides tumor-stroma cross-talk, the colonic fibroblast secretome and senescence-associated secreted phenotype (SASP) play a crucial role in regulating the proliferation of cancer cells [11, 28]. Secreted factors from normal and senescent stromal cells have also been suggested to contribute to tumorigenesis and differential drug effects [29].

Since the advanced-stage colon cancers spread from primary tumor site to distant organs and tissues [30], the colon-unassociated stromal population may play an important role in forming a favorable metastatic niche for CRC cells. The interactions between CRC cells and noncolon fibroblasts at the distant metastatic sites are important, yet these heterocellular tumor-stroma interactions for preventive and/or therapeutic strategies for metastatic colorectal cancer remain understudied. In this study, we investigated the effect of colon-unassociated normal human foreskin and lung fibroblasts and their radiation-induced senescent counterparts on CRC cell response to AZA and DAC in two-dimensional (2D) and spheroid cultures. In addition, we studied the effect of conditioned medium from normal and senescent fibroblasts cells on colon cancer cell proliferation and DAC-induced DNA demethylation. This study was performed using our recently described demethylation reporter, HCT116-pFLJ-H2B cells, henceforth referred to as HCT116 [31].

## 2. Materials and Methods

### 2.1. Chemicals, Cell Culture, and Reporter Cells

AZA and DAC were synthesized as described previously [32]. DMSO concentration was always less than 0.1% in treated wells.

Human normal BJ foreskin fibroblasts (ATCC® CRL-2522™) and human normal MRC-5 lung fibroblasts (ATCC CCL-171™) were purchased from ATCC (Middlesex, UK) and cultured in EMEM (Gibco®, Thermo Fisher Scientific Inc., Waltham, MA, USA) supplemented with 10% fetal bovine serum (FBS; Gibco, Thermo Fisher Scientific). Human A549 lung carcinoma cells (ATCC CCL-185™) were cultured in Ham's F-12 medium (Gibco, Thermo Fisher Scientific) supplemented with 10% FBS. All cells were maintained in a standard humidified incubator in 5% CO_2_/atmospheric air at 37°C.

Demethylation reporter HCT116 cells were generated and cultured as described previously [31]. GFP-expressing BJ cells (BJ-GFP) were generated by transduction using Cignal Lenti GFP lentiviral particles, whereas nuclear factor-*κ*B (NF-*κ*B) reporter A549 cells (A549-NF-*κ*B) were generated using Cignal Lenti NF-*κ*B Reporter lentiviral particles from Qiagen (Hilden, Germany) following the manufacturer's protocol. Briefly, all cells were infected at a multiplicity of infection of 10 pfu/cell. To enhance the efficiency of transduction, SureENTRY Transduction Reagent (Qiagen) was used at a concentration of 8 *μ*g/mL. Transduced cells were subjected to selection pressure of 3 *μ*M puromycin (Sigma-Aldrich, St. Louis, MO, USA). BJ-GFP cells were isolated by single-cell sorting in a BD FACSAria II cell sorter (BD Biosciences, San Jose, CA, USA) in order to avoid multiple passages and replicative senescence during clonal selection.

### 2.2. Senescence Induction by X-Ray Irradiation, Conditioned Medium, and Cell Viability Assay

The fibroblast cultures were exposed to 10 Gy X-ray irradiation in an X-ray RS225 irradiator (Xstrahl, Surrey, UK) at a dose rate of 2.3 Gy/min. Irradiated cells were then maintained for 1 to 3 weeks before the collection of conditioned medium or the use of cells for experiments. Collected conditioned medium was filtered using a 0.22 *μ*m sterile syringe filter (Merck Millipore, Burlington, MA, USA) and diluted to 25% in the complete fresh medium before experiments to provide the vital components necessary to support the cell growth.

For cell viability assays, HCT116 were seeded in 96-well plates and exposed to X-ray irradiation as described above. After 8 h, irradiated HCT116 were treated with DAC (0.2–20 *μ*M) either in 25% conditioned medium from irradiated BJ or complete medium for 72 h, and cell viability was determined by a standard 3-(4,5-dimethylthiazol-2-yl)-2,5-diphenyltetrazolium bromide (MTT) assay.

### 2.3. *β*-Galactosidase Assay for Senescent Cells

Senescent cells in nonirradiated and 1- to 3-week-old irradiated fibroblast cultures were stained using a *β*-Galactosidase (*β*-Gal) Staining Kit (Cell Signaling Technology, Danvers, MA, USA) following the manufacturer's protocol. Cells were counterstained with Hoechst 33342 (Molecular Probes®, Eugene, OR, USA) prior to imaging in a Cell Voyager CV7000S microscope (Yokogawa, Tokyo, Japan) using a 20x objective and 405/488/561 nm laser line (Hoechst) and bright field filter for *β*-Gal. Captured images were imported to Columbus™ Image Analysis System (PerkinElmer, Waltham, MA, USA). Senescent cells were quantified using a texture-based analysis of the nuclear and cytoplasmic regions by a Saddle-Edges-Ridges (SER) algorithm in Columbus Image Analysis System [33]. Briefly, cell nuclei were identified based on Hoechst staining. Then, the area and roundness of identified nuclei were calculated, and cell population was selected based on area and roundness. Next, the cytoplasm around the selected population of nuclei was identified to calculate the texture properties (in bright field channel) based on SER spot features. Cells with SER spot value higher than the threshold value were quantified and calculated.

To analyze protein markers of cellular senescence, replicating and senescent fibroblasts were collected and processed for Western blot analysis as described below.

### 2.4. Coculture and Conditioned Medium Culture Setup

Monocultures of HCT116 and cocultures of HCT116 and nonirradiated or 1- to 3-week-old irradiated senescent fibroblasts were established in clear-bottom CellCarrier 384-well plates (PerkinElmer) at 7 : 3 and 3 : 7 ratios, hereafter referred to as low stromal cocultures and high stromal cocultures, respectively. The total cell density was always 1000 cells/well. Note that all cocultures were established in EMEM that supported the normal growth of all cell types.

For studying the effects of conditioned medium from senescent fibroblast cultures on HCT116 proliferation and demethylation, the experiment was set in a way that there was a free exchange of medium between HCT116 cells and 1- to 3-week-old irradiated fibroblasts in different wells in the absence of a direct cell-to-cell contact.

### 2.5. Drug Treatment, Demethylation, and Cell Proliferation Analysis in 2D Cultures

Cells were treated for 72 h with DAC or AZA at 1 *μ*M and 5 *μ*M concentrations diluted in appropriate medium and imaged and analyzed to evaluate the intensity of EGFP signal as described elsewhere [31]. The rate of HCT116 cell proliferation (72 h/24 h) in untreated culture types was determined by counting the total number of RFP-H2B-tagged HCT116 cell nuclei using Columbus Image Analysis System (PerkinElmer), as described previously [31].

### 2.6. Spheroid Culture, Drug Treatment, and Imaging

Spheroids were generated as described elsewhere [34]. Low stromal coculture and high stromal coculture spheroids of HCT116 and nonirradiated or 1- to 3-week-old irradiated fibroblasts were established at ratios described above (see Section 2.4). Spheroids were grown for at least 1 week before the start of any treatment. To study the effect of conditioned medium, spheroids were transferred to a new agarose-coated 384-well plate containing 25% conditioned medium from irradiated fibroblast cultures. Spheroid imaging and quantification of EGFP intensity and spheroid size were carried out as described elsewhere [31, 34]. All drug treatments in spheroids were done for 96 h.

Images of DAC-treated spheroids were acquired using a Light Sheet Z.1 microscope (Carl Zeiss, Jena, Germany). Prior to imaging, DAC-treated spheroids were collected and washed in 1x phosphate-buffered saline (PBS). Spheroids were then stained for 2 h with 10 *μ*M Hoechst nuclear dye at room temperature. Spheroids were washed in 1x PBS to remove residual Hoechst and mounted in 1.5% (*w*/*v*) low-melting agarose (40°C). Spheroids were then drawn into a 0.5 mm glass capillary tube with a metal plunger (Carl Zeiss) and allowed to polymerize for 5 min at room temperature. The capillary tube was then vertically mounted on a sample holder and immersed in a sample chamber filled with phenol-red free EMEM. The polymerized agarose containing spheroids was then extruded into the sample chamber using the metal plunger, and multidirectional *z*-stack images were acquired using a 20x detection optics and two 10x illumination optics with appropriate lasers and filters. The captured images were processed using ZEN Blue image processing software (Carl Zeiss).

### 2.7. Cell Sorting and Western Blot Analysis

HCT116 cultured in 25% conditioned medium from nonirradiated or 1- to 3-week-old irradiated fibroblast cultures and high stromal cocultures of HCT116 with nonirradiated or irradiated fibroblasts were treated with 1 *μ*M DAC for 72 h. HCT116 from conditioned medium cultures were collected and immediately lysed and processed for Western blot analysis following drug treatment. To analyze the effect of DAC on HCT116 in cocultures, RFP-expressing HCT116 were first isolated by sorting in a FACSAria II Cell Sorter (BD Biosciences) and then processed for Western blotting. Nonfluorescent normal BJ and sBJ cells from cocultures were also isolated simultaneously for Western blot experiments.

Cells were lysed in RIPA buffer (150 mM NaCl, 1.0% NP-40 or Triton X-100, 0.5% sodium deoxycholate, 0.1% sodium dodecyl sulfate, 50 mM Tris, (pH 8.0)) supplemented with cOmplete™ Protease Inhibitor Cocktail (Roche Holding AG, Basel, Switzerland) by sonication on ice. Protein lysates (20–50 *μ*g) were electrophoresed and transferred onto a PVDF membrane (Merck Millipore) and probed with antibodies as described elsewhere [35]. Primary antibodies against DNA methyltransferase 1 (DNMT1; catalogue number: 5032, 1 : 1000 dilution), vimentin (catalogue number: 5741, 1 : 1000 dilution), *β*-catenin (catalogue number: 8480, 1 : 1000 dilution), and p21^waf1/cip^ (catalogue number: 2947, 1 : 1000 dilution) were purchased from Cell Signaling Technology (Danvers, MA, USA); p53 (catalogue number: ab131442, 0.02 *μ*g/mL dilution) from Abcam (Cambridge, UK); p16 (catalogue number: sc-759; 1 : 500 dilution) from Santa Cruz Biotechnology (Dallas, TX, USA); and Tet methylcytosine dioxygenase 1 (TET1; catalogue number: NBP2-15135; 1 : 1000 dilution) and deoxycytidine kinase (dCK; catalogue number: H00001633-B01P; 1 *μ*g/mL dilution) from Novus Biologicals (Littleton, CO, USA). Mouse anti-*β*-actin antibody (catalogue number: A5441; 1 : 4000 dilution) was used as a loading control and was purchased from Sigma-Aldrich. Blots were developed using either goat anti-mouse or anti-rabbit Alexa Fluor® 488 secondary antibodies (1 : 2000 dilution) from Life Technologies (Carlsbad, CA, USA).

### 2.8. NF-*κ*B Activity and Cytokine Assays

To determine NF-*κ*B activity, in-house developed A549-NF-*κ*B reporter cells [36] were seeded at a density of 10,000 cells/well in Ham's F-12 medium in white opaque 96-well plates (PerkinElmer). After 24 h, the old medium was replaced with undiluted conditioned medium from nonirradiated or 1- to 3-week-old irradiated fibroblast cultures, and the cells were further incubated for 24 and 48 h. At the end of each incubation, 100 *μ*L Britelite Plus luminescent reagent (PerkinElmer) was added per well, plate content was mixed in a plate shaker, and the luminescent signal was measured in an EnVision Multilabel Plate Reader (PerkinElmer).

The proinflammatory cytokines and/or chemokines in conditioned medium were assayed using a Cytokine Human Magnetic 25-Plex Panel Luminex™ Kit (Life Technologies) following the manufacturer's protocol and analyzed in a Luminex 200 System Analyzer (Austin, TX, USA).

All assays were performed with samples of condition medium obtained from three independent cultures of nonirradiated or irradiated fibroblast cultures.

### 2.9. Statistical Analysis

All statistical analyses were performed on at least 2–4 independent biological replicates using GraphPad Prism (GraphPad Software version 7, San Diego, CA, USA), and differences were considered significant at *p* < 0.05. Unless otherwise mentioned, data were analyzed using one-way ANOVA with Dunnett's multiple comparison test. For one-sample *t*-test, data were compared with a hypothetical value of 100%.

## 3. Results

### 3.1. Irradiation Increased the Number of *β*-Gal-Positive Senescent Fibroblast Cells

Irradiation is a well-reported inducer of senescence in different cell types [13]. Therefore, we first determined the number of *β*-Gal-stained senescent cells in nonirradiated and 1-week-old irradiated BJ fibroblast cultures by high content image analysis as described in Materials and Methods. Compared to nonirradiated BJ cultures, there was a significant increase in the number of *β*-Gal-positive senescent BJ (sBJ) fibroblasts post 1 week of irradiation (Figure S1a; 2.7 ± 0.3% in nonirradiated BJ versus 5.5 ± 0.5% in 1-wk-IR sBJ cultures, *p* < 0.001, *n* = 2, Student's *t*-test, unpaired). Culturing the irradiated BJ fibroblasts for an additional 2 weeks further increased the percentage of *β*-Gal-positive cells to 42.9 ± 3.1% (*p* < 0.001 versus nonirradiated BJ, *n* = 2, Student's *t*-test, unpaired).

Next, we determined the induction of molecular markers of senescence in nonirradiated and 1- to 3-week-old irradiated BJ cultures. The nonirradiated BJ fibroblasts showed a weakly elevated level of p21^waf1/cip^. In accordance with the *β*-Gal staining data, irradiation induced the expression of senescence markers, p16 and p21^waf1/cip^, in addition to p53, in 1- to 3-week-old sBJ cultures (Figure S1b).

### 3.2. Fibroblasts Increased the Susceptibility of HCT116 to DAC in 2D Cocultures

**Figure 1:**
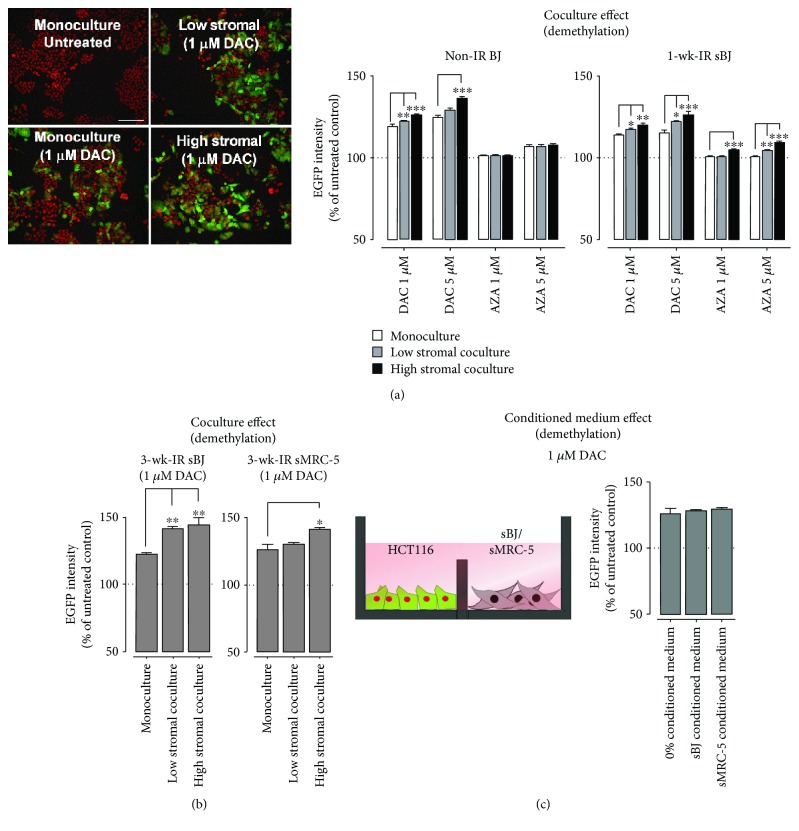
Effect of fibroblasts and their conditioned medium on HCT116 DNA demethylation in 2D cultures. (a) Representative images showing RFP nuclear fluorescence but no EGFP fluorescence in the untreated control, and changes in EGFP fluorescence following DAC treatment in HCT116 monocultures or cocultures with BJ. 20x objective; scale bar: 100 *μ*m. Graphs showing a significant increase in EGFP intensity in HCT116 cocultured with normal nonirradiated (non-IR) BJ and 1-week-irradiated (1-wk-IR) sBJ fibroblasts in comparison to HCT116 monocultures following treatment with different concentrations of DAC and AZA. Data are mean ± SEM, *n* = 2–4, ^∗∗^ *p* < 0.01, ^∗^ *p* < 0.05 compared to HCT116 monocultures. (b) A significant increase in EGFP intensity of HCT116 cells cocultured with 3-week-irradiated (3-wk-IR) sBJ or sMRC-5 cells following treatment with 1 *μ*M DAC. (c) Schematic diagram of the setup of conditioned medium culture and graph showing no effect of sBJ or sMRC-5 conditioned medium on EGFP intensity following treatment of HCT116 monocultures with 1 *μ*M DAC. Data are mean ± SEM, *n* = 2–4.

To examine the effect of senescent fibroblasts on HCT116 response to DNA-demethylating drugs, low and high stromal cocultures of HCT116 and 1-week-old irradiated sBJ were established. A comparison of EGFP intensities showed a culture-dependent increase in the effect of DAC and AZA on HCT116 DNA demethylation in the order of high stromal coculture > low stromal coculture > monoculture (Figure 1(a)). To examine if this effect was limited to senescent cells, we performed a similar comparison following DAC and AZA treatment of HCT116 in coculture with nonirradiated BJ fibroblasts. Similar to sBJ fibroblasts, the presence of nonirradiated BJ fibroblasts significantly increased DAC-induced HCT116 DNA demethylation, but there was no difference in the effect of AZA (Figure 1(a)). Since DAC had a greater effect on HCT116 demethylation in cocultures, we decided to perform all subsequent studies with DAC. Also, as evident from the previous study conducted in HCT116 cells, DAC showed maximum demethylation at 1 *μ*M concentration; therefore, we chose 1 *μ*M DAC concentration for the further studies [37]. Next, to see if the observed senescent cell effect was reproduced by other senescent fibroblast types, we treated cocultures of HCT116 and 3-week-old irradiated senescent MRC-5 (sMCR-5) and sBJ fibroblasts with 1 *μ*M DAC. The data showed an increased demethylation effect of DAC on HCT116 in coculture with both sMRC-5 and 3-week irradiated sBJ fibroblasts (Figure.  1(b)). Overall, the data indicate that the increase in demethylation of HCT116 by DNMTIs is more pronounced in the presence of senescent fibroblasts.

We next examined the effect of SASP on HCT116 response towards DAC-induced demethylation. Cells were cultured in a way that there was a free exchange of medium between HCT116 and sBJ or sMRC-5 cells, but there was no direct HCT116 to sBJ or sMRC-5 cell-cell contact (Figure 1(b)). The results showed no significant effect of SASP on 1 *μ*M DAC-induced demethylation in HCT116 (Figure 1(b)).

### 3.3. Fibroblasts and Their Conditioned Medium Affect DAC-Induced Alteration in DNA Methyltransferase 1 Level

**Figure 2:**
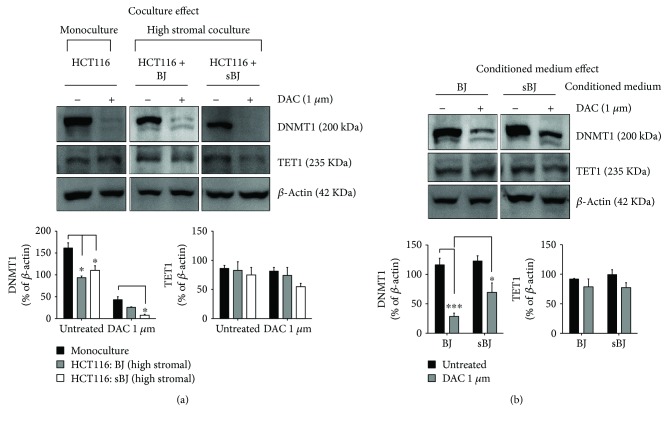
Changes in the expression of DNMT1 and TET1. Representative western blots and densitometry analysis of DNMT1 and TET1 levels in untreated or DAC-treated (a) HCT116 monocultures and cocultures with normal BJ or sBJ and (b) HCT116 monocultures grown in conditioned medium from normal BJ or sBJ cells. DNMT1 and TET1 blots in (a) and (b) are taken from different gels. Data are mean ± SEM, *n* = 3, ^∗∗∗^ *p* < 0.001, ^∗^ *p* < 0.05.

To further decipher the effect of normal and senescent fibroblasts, we analyzed the changes in the protein levels of DNA methylation and demethylation-regulating enzymes, DNMT1 and TET1, respectively, in HCT116 isolated from cocultures of HCT116 and nonirradiated BJ or sBJ fibroblasts. The results showed significant downregulation of DNMT1 in untreated HCT116 that were cocultured with nonirradiated BJ or sBJ fibroblasts compared to HCT116 monocultures. Although 1 *μ*M DAC downregulated DNMT1 levels in monoculture HCT116, the downregulation was significantly greater in HCT116 cocultured with sBJ (Figure 2(a); *p* = 0.03, two-way ANOVA). Although there were alterations in the level of TET1 in HCT116 following DAC treatment in different culture types, the difference was statistically nonsignificant (Figure 2(a)).

We further studied the effect of conditioned medium from nonirradiated BJ and sBJ fibroblasts on DNMT1 and TET1 levels in HCT116. While 1 *μ*M DAC inhibited DNMT1 levels in HCT116 when the treatment was done in the presence of conditioned medium from normal BJ fibroblasts, this inhibition was abrogated in the presence of conditioned medium from sBJ cultures (Figure 2(b)). The data relates to the lack of significant increase in DAC-induced HCT116 DNA demethylation in the presence of conditioned medium from sBJ cultures (see Figure 1(b)). There was no significant difference in TET1 levels in HCT116 cells in condition medium cultures (Figure 2(b)).

Radiation elevates dCK mRNA and protein levels [38], and there is a clear correlation between dCK levels and radiosensitizing effects of gemcitabine [39]. dCK adds the first phosphoryl group to DAC and is the rate-limiting enzyme of the overall process of converting DAC to its triphosphate form that incorporates into DNA [40]. We next examined the protein levels of dCK in DAC-treated HCT116 isolated from cocultures with BJ or sBJ fibroblasts and those cultured in conditioned medium from BJ or sBJ cultures. Indeed, our data revealed an elevated level of dCK in DAC-untreated HCT116 isolated from cocultures of HCT116 with BJ or sBJ fibroblasts; however, this expression was higher in HCT116 isolated from cocultures with sBJ fibroblasts (Figure S2a). The treatment with DAC seemed to further increase the levels of dCK in coculture-isolated HCT116. Interestingly, the presence of conditioned medium from sBJ cultures reduced the level of dCK in HCT116 treated with or without DAC (Figure S2b).

### 3.4. Fibroblasts and their Conditioned Medium Increased Cell Proliferation in 2D Cultures

**Figure 3:**
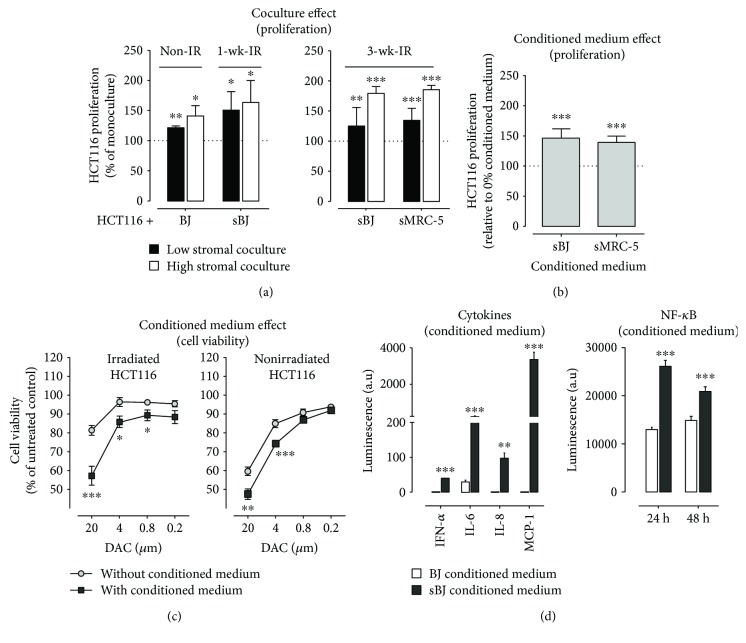
Coculture effect on HCT116 proliferation and analysis of secretory factors in conditioned medium. (a, b) The effect of normal and senescent fibroblasts (a) and SASP of senescent fibroblasts (b) on the proliferation of HCT116. Data are mean ± SEM, *n* = 2–4, ^∗∗∗^ *p* < 0.001, ^∗∗^ *p* < 0.01, ^∗^ *p* < 0.05 compared to HCT116 monocultures or 0% conditioned medium, one-sample *t*-test. (c) Viability of nonirradiated and irradiated HCT116 following DAC treatment. Data are mean ± SEM, *n* = 4, ^∗∗∗^ *p* < 0.001, ^∗∗^ *p* < 0.01 comparing cell viability with or without conditioned medium, Student's *t*-test, unpaired. (d) Increased levels of IFN-*α*, IL-6, IL-8, and MCP-1 in sBJ conditioned medium compared to conditioned medium from normal BJ cells and its effect on NF-*κ*B activity. Data are mean ± SEM, *n* = 3, ^∗∗∗^ *p* < 0.001, ^∗∗^ *p* < 0.01 comparing conditioned medium from BJ to sBJ, Student's *t*-test, unpaired.

Since the demethylation by DAC is more pronounced in proliferating cells [41], we examined the effects of normal and senescent fibroblasts and their conditioned medium on HCT116 proliferation. Compared to monocultures, HCT116 proliferation was markedly increased when cocultured with either normal, sBJ, or sMRC-5 fibroblasts or in the presence of conditioned medium from senescent fibroblasts (Figures 3(a) and 3(b)).

### 3.5. Senescent Fibroblast Conditioned Medium Increased DAC-Induced Cytotoxicity and Displayed High Levels of Proinflammatory Cytokines and Chemokines

To determine if senescent fibroblast conditioned medium-induced increase in the proliferation of HCT116 was partly responsible for increasing the susceptibility of HCT116 to DAC, we next determined the cytotoxic/cytostatic effects of DAC in nonproliferating HCT116 in the presence of conditioned medium. We first irradiated HCT116 to induce cell cycle arrest [42] and then treated irradiated HCT116 with DAC (0.2–20 *μ*M) in the absence or presence of conditioned medium from sBJ cultures. Irradiation of HCT116 attenuated the cytotoxic/cytostatic effect of DAC in the absence of conditioned medium; however, the addition of sBJ conditioned medium reversed this effect (Figure 3(c), left). Although DAC was significantly effective in altering the viability of nonirradiated HCT116, the addition of conditioned medium further increased DAC effect (Figure 3(c), right).

The NF-*κ*B pathway is suggested to contribute to senescence program [43], and DNA-demethylating agents induce apoptosis by inhibiting NF-*κ*B activity [44]. Evidence also suggests a correlation between NF-*κ*B and DNMT1 levels [45]. Besides, a recent study showed the role of inflammatory cytokines in regulating the activity of enzymes involved in DNA methylation and demethylation [46]. Given this correlation, we analyzed the levels of a panel of 25 human cytokines and chemokines in conditioned medium from normal BJ and sBJ cultures and the effect of condition medium on NF-*κ*B activity. Conditioned medium from sBJ showed a high level of interferon-alpha (IFN-*α*), interleukin-6 (IL-6) and interleukin-8 (IL-8), and monocyte chemotactic protein-1 (MCP-1) compared to conditioned medium from normal BJ cultures (Figure 3(d)). The conditioned medium from sBJ significantly increased NF-*κ*B activity in NF-*κ*B reporter cellular model (Figure 3(d)).

### 3.6. Increased Susceptibility of HCT116 to DAC in Coculture Spheroids

**Figure 4:**
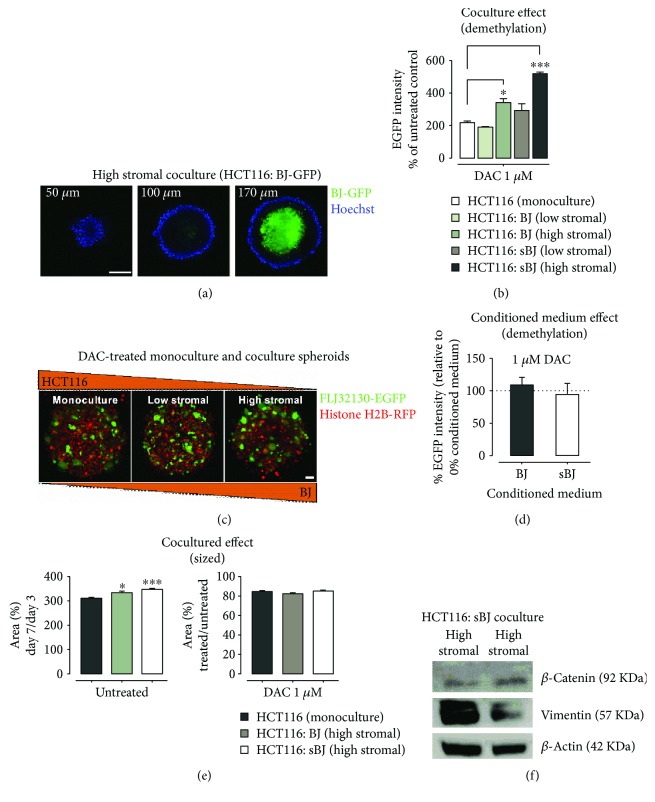
Fibroblasts and conditioned medium-induced effects on HCT116 susceptibility to DAC in spheroid cultures and expression of EMT markers. (a) Images of high stromal cell coculture spheroids showing the presence of BJ cells in the spheroid interior. The *z*-plane heights are indicated on the top of images. HCT116 are nonfluorescent and the exterior of the spheroid is stained blue with Hoechst. 20x objective; scale bar: 50 *μ*m. (b) Increase in EGFP intensity in coculture spheroids of HCT116 and BJ or sBJ cells compared to HCT116 monoculture spheroids. Data are mean ± SEM, *n* > 20 spheroids per group from 3 independent experiments, ^∗∗∗^ *p* < 0.001, ^∗^ *p* < 0.05, Kruskal-Wallis test with Dunnet's multiple comparisons test. (c) Representative images showing the effect of DAC on HCT116 monoculture spheroids and coculture spheroids of HCT116 and BJ fibroblasts. 20x objective; scale bar: 10 *μ*m. (d) The effect of conditioned medium from normal and sBJ cultures on DAC-induced demethylation of HCT116 monoculture spheroids. Data are mean ± SEM, *n* > 20 spheroids per group from 3 independent experiments, one-sample *t*-test. (e) Increase in the size of untreated coculture spheroids of HCT116 and BJ or sBJ compared to HCT116 monoculture spheroids (left) and no effect of DAC treatment on coculture spheroid size (right). Data are mean ± SEM, *n* > 20 spheroids per group from 3 independent experiments, ^∗∗∗^ *p* < 0.001, ^∗^ *p* < 0.05, Kruskal-Wallis test with Dunnet's multiple comparison test. (f) Representative Western blots showing the induction of vimentin expression in HCT116 sorted from high and low stromal cell coculture spheroids of HCT116 and sBJ.

Cell-cell interactions in spheroids are closer to physiological conditions, and therefore spheroids are excellent models to study the effect of tumor-stroma interaction on tumor cell response to anticancer drugs. We next investigated the effect of normal BJ and sBJ fibroblasts on HCT116 DNA demethylation in coculture spheroids following 1 *μ*M DAC treatment. First, despite the cell number, BJ fibroblasts always occupied the center of spheroids surrounded by HCT116. The GFP-expressing BJ fibroblasts were visible only after approximately 100 *μ*m *z*-plane height (Figure 4(a)), indicating a limited stromal-tumor cell contact and underlying the importance of autocrine and/or paracrine factors. Similar to 2D cultures, the presence of a high number of normal BJ or sBJ cells increased HCT116 demethylation in coculture spheroids (Figures 4(b) and 4(c)); however, this effect was more pronounced in sBJ fibroblast-containing spheroids (Figure 4(b)).

Next, to determine the effect of conditioned medium on DAC-induced HCT116 demethylation, we treated monoculture spheroids of HCT116 with 1 *μ*M DAC in the presence of conditioned medium from normal BJ and sBJ cultures. There was no significant effect of sBJ conditioned medium on DAC-induced demethylation on monoculture spheroids (Figure 4(d)).

Given the fact that DAC is more effective in proliferating cells [41], we determined whether the increased demethylation in coculture spheroids is related to the increased growth of spheroids. We compared the size of HCT116 monoculture spheroids with coculture spheroids of HCT116 and normal BJ or sBJ fibroblasts. The results showed a significant increase in the size of coculture spheroids compared to monoculture spheroids (Figure 4(e)). However, there was no major difference in the effect of 1 *μ*M DAC on coculture spheroid size compared to monoculture spheroids (Figure 4(e)).

A recently published study elucidated that although cellular senescence arrests cell cycle program, the key signaling components of the senescence machinery, such as p16, p21^waf1/cip^, and p53, critically regulate stem cell functions and promote stemness of cancer cells [47]. Therefore, we examined the protein expression levels of vimentin, a typical phenotype of EMT, and activation of Wnt/*β*-catenin in HCT116 sorted from coculture spheroids of HCT116 and sBJ. The results demonstrated an upregulated expression of vimentin in HCT116 from high stromal coculture spheroids (Figure 4(f)). The results (Figure S1 and Figure 4(f)) relate increased growth of coculture spheroids to sBJ-induced stemness in HCT116.

## 4. Discussion

Studies indicate potential synergistic effects of DNMTIs and radiotherapy for the treatment of solid tumors [26, 27]. Given the senescence-inducing property of radiation, it remains to be seen whether and/or how the senescent stromal cells affect tumor cell response to DNMTIs. Using our recently developed DNA demethylation reporter cells [31], we show that senescent fibroblasts increase the demethylation effects of DAC in HCT116 under coculture conditions in both 2D and spheroid cultures (Figures 1 and 4). Furthermore, the increased DNA demethylation in high stromal cocultures than monocultures suggests the increased susceptibility of HCT116 to DAC in a higher stromal microenvironment. The increased demethylation effect of DAC was not just limited to cocultures containing senescent fibroblasts as the presence of nonirradiated normal fibroblast also induced a similar effect, albeit smaller, on HCT116 DNA demethylation in both 2D and spheroid cultures. Nevertheless, the demethylation effect was more pronounced in cocultures of HCT116 with irradiation-induced senescent fibroblasts that showed increased expression of p21^waf1/cip^ and p16 (Figure S1, Figures 1 and 4). Repeated subculturing has been reported to induce replicative senescence in fibroblasts [48]. The increased HCT116 DNA demethylation in cocultures with nonirradiated fibroblasts could have presumably resulted due to the presence of presenescent fibroblasts. This is evident from the presence of a small fraction of *β*-Gal-stained cells and expression of p21^waf1/cip^ in nonirradiated BJ cells (Figure S1).

Our data also demonstrate the fibroblast-induced downregulation of endogenous levels of DNMT1 in untreated HCT116. Additionally, the data also show the increased effect of DAC on DNMT1 levels in HCT116 sorted from cocultures than monocultures (Figure 2). Exposing cancer cells to gamma irradiation has been reported to decrease the protein levels of DNMT1 and DNMT3b [49, 50]. Further, studies indicate that activation of nucleoside analogs correlates with dCK activity [39, 51]. We show an elevation of dCK protein levels in HCT116 cocultured with fibroblasts, in particular, irradiation-induced sBJ cultures (Figure S2). This increase in dCK levels corresponds to the increased demethylation effect of DAC on HCT116 in high stromal cocultures (Figure 1). Overall, the data indicate the potential role of radiation-induced bystander effect through tumor-stroma cross-talk in regulating epigenetic changes in tumor cells in high stromal cocultures.

The stroma has been reported to regulate the growth of tumor cells, increasing their invasive and metastatic properties [10]. In line with this, we observed normal and senescent fibroblast-induced increased proliferation of HCT116 in 2D and spheroid cocultures (Figures 3 and 4). Since DAC, like other anticancer drugs, is reported to have a greater effect in actively proliferating cells [41], the increased proliferation of HCT116 in both 2D and spheroid cocultures potentially makes HCT116 more susceptible to DAC (Figures 3 and 4). This is partly shown by the decreased effect of DAC on the viability of irradiated HCT116 (Figure 3).

Apart from fibroblast-induced effects in cocultures, we also studied the effect of conditioned medium from senescent fibroblasts on HCT116 proliferation in 2D cultures. The results demonstrated an increased effect of conditioned medium from senescent fibroblast cultures on HCT116 proliferation only. However, conditioned medium from senescent fibroblasts abrogated DAC effect on DNMT1 expression in treated cells and decreased dCK levels in untreated and DAC-treated HCT116 (Figure S2). Analysis of conditioned medium from senescent fibroblasts showed upregulation of proinflammatory cytokine and chemokine levels. This cytokine/chemokine-laden condition medium increased NF-*κ*B activity in NF-*κ*B A549 reporter cells. The correlation between demethylation effects of DNMTIs and NF-*κ*B remains debatable in the literature. While one study suggests that the apoptosis induced by DNMTIs via inhibition of NF-*κ*B is not due to epigenetic reprogramming [44], another study showed that an increase in NF-*κ*B activity downregulates DNMT1 levels [45]. In our study, we did not observe any direct effect of senescent fibroblast conditioned medium on the protein levels of DNMT1 (Figure 2). Nonetheless, the inability of DAC to reduce DNMT1 levels in cells treated in conditioned medium from senescent fibroblast cultures (Figure 2) indicates the potential negative effect of SASP on DNA demethylation. The present study was conducted using established cell line cultures only; therefore, a further line of evidence from primary cells and DNMT1 knockout cell types is required. Also, secretome analysis is clearly required to substantiate the correlation between NF-*κ*B activity and/or proinflammatory cytokines and chemokines on DNA methylation and demethylation.

## 5. Conclusions

In agreement with the prognostic significance of tumor-stroma ratio in different cancer types, the results of our study indicate the potential of the tumor-stroma ratio for predicting the outcome of DNA-demethylating epigenetic anticancer therapy in CRC or other cancer types. The study further correlates the increased susceptibility of HCT116 to DAC due to fibroblast-induced increased proliferation and differential regulation of methylation- and demethylation-regulating enzymes by senescent stromal cells. In conclusion, this study provides the evidence of the senescent stromal cell-induced effects on CRC cell response towards prototypal DNA-demethylating drug, DAC. Further studies are required to confer the mechanism behind observed stromal cell-induced alterations in DAC-induced DNA demethylation effects.

## Data Availability

The data used to support the findings of this study are available from the corresponding author upon request.

## Supplementary Materials

Supplementary Materials Figure S1: radiation increased senescence in fibroblast cultures. (a) Representative images showing Hoechst- and *β*-Gal-stained cultures of nonirradiated (non-IR) and 1- to 3-week-old irradiation- (IR-) induced senescent BJ (sBJ) cultures. The values in the middle *β*-Gal panel show the percentage of *β*-Gal positive cells. Data are mean ± SEM, *n* > 10 wells per 384-well plate from 2 independent experiments. 20x objective; scale bar: 100 *μ*m. (b) Western blots showing the expression of p53, p21^waf1/cip^_,_ and p16 in nonirradiated (non-IR) BJ and 1- to 3-week-old sBJ cells following irradiation (IR). Figure S2: coculture and conditioned medium effect on dCK expression. (a) Western blots showing changes in dCK expression in BJ and HCT116 isolated from cocultures of HCT116 and normal and sBJ cells in the absence (−) or presence (+) of DAC. (b) Changes in the expression of dCK in DAC-treated HCT116 cultured in conditioned medium from BJ or sBJ cultures. The fold expression of dCK relative to *β*-actin loading control is shown below the blots in (a) and (b) from one experiment.
