# Supplementary material for: Differential Regulation of Methylation-Regulating Enzymes by Senescent Stromal Cells Drives Colorectal Cancer Cell Response to DNA-Demethylating Epi-Drugs
Source: Stem Cells Int. 2018 Aug 12;2018:6013728. doi: 10.1155/2018/6013728 (PMC6109465; doi:10.1155/2018/6013728)
Supplement: Supplementary Materials — Figure S1: radiation increased senescence in fibroblast cultures. (a) Representative images showing Hoechst- and β-Gal-stained cultures of nonirradiated (non-IR) and 1- to 3-week-old irradiation- (IR-) induced senescent BJ (sBJ) cultures. The values in the middle β-Gal panel show the percentage of β-Gal positive cells. Data are mean ± SEM, n > 10 wells per 384-well plate from 2 independent experiments. 20x objective; scale bar: 100 μm. (b) Western blots showing the expression of p53, p21waf1/cip , and p16 in nonirradiated (non-IR) BJ and 1- to 3-week-old sBJ cells following irradiation (IR). Figure S2: coculture and conditioned medium effect on dCK expression. (a) Western blots showing changes in dCK expression in BJ and HCT116 isolated from cocultures of HCT116 and normal and sBJ cells in the absence (−) or presence (+) of DAC. (b) Changes in the expression of dCK in DAC-treated HCT116 cultured in conditioned medium from BJ or sBJ cultures. The fold expression of dCK relative to β-actin loading control is shown below the blots in (a) and (b) from one experiment. [file 6013728.f1.docx]

# Supplementary figures

# Title

Differential regulation of methylation-regulating enzymes by senescent stromal cells drives colorectal cancer cell response to DNA epi-drug decitabine

**Authors and Affiliations**

Khushboo Agrawal,^1, 2^ Viswanath Das,^1, 2^ Natálie Táborská,^1^ Ján Gurský,^1^ Petr Džubák,^1, 2^ Marián Hajdúch^1, 2^

^1^ Institute of Molecular and Translational Medicine, Faculty of Medicine and Dentistry, Palacky University, Hněvotínská 5, 77900 Olomouc, Czech Republic

^2^ Foundation for Cancer Research Czech Republic, Hněvotínská 5, 77900 Olomouc, Czech Republic

*Both Khushboo Agrawal and Viswanath Das contributed equally to this work*

## Corresponding author

Marián Hajdúch , M.D., Ph.D (Email: [marian.hajuch@upol.cz](mailto:marian.hajuch@upol.cz), Tel.: +420 603 552 111, Fax: +420 585 632 180)

**Figure S1**

**
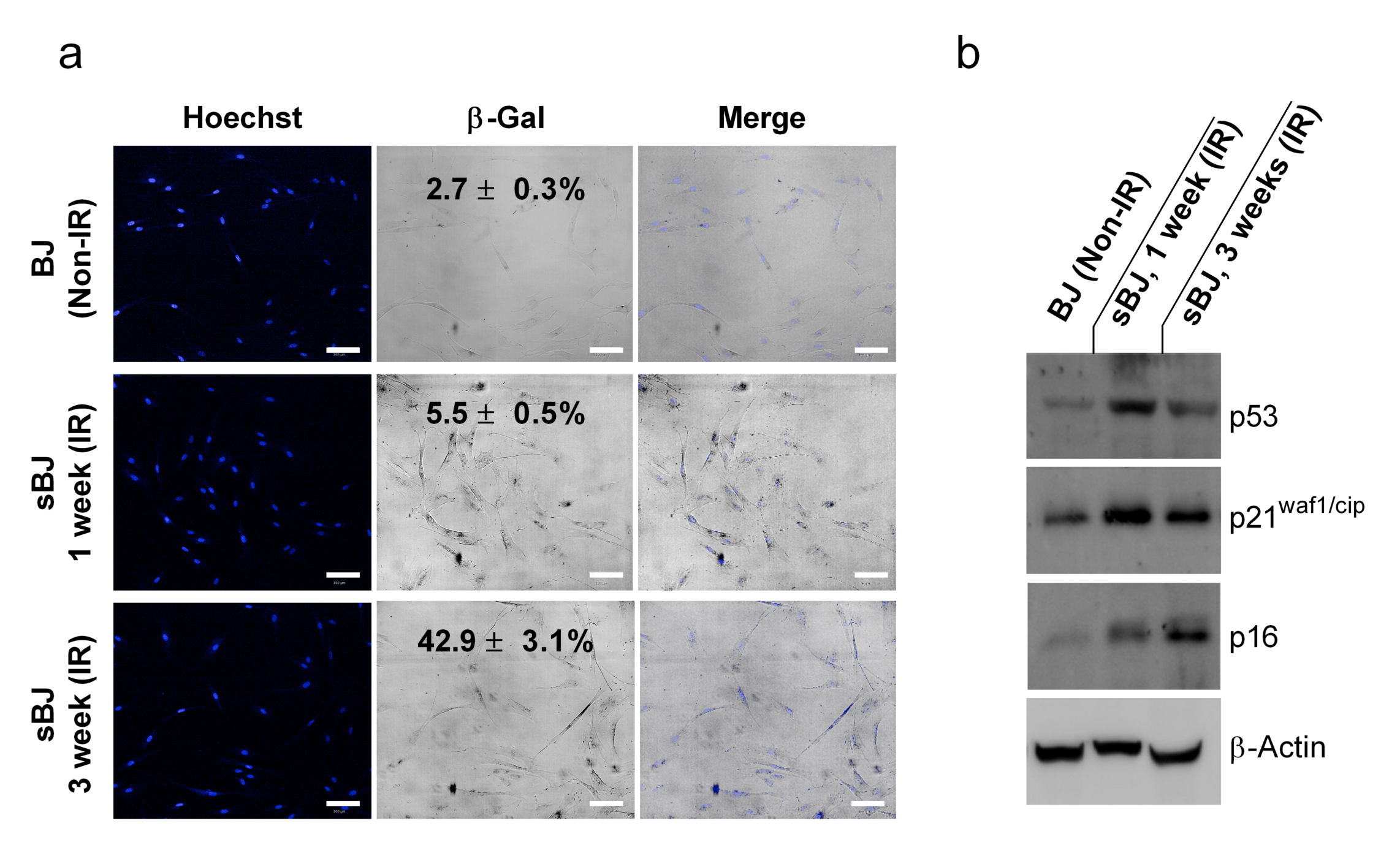
**

**Figure S1. Radiation increased senescence in fibroblast cultures.** (a) Representative images showing Hoechst- and β-Gal-stained cultures of non-irradiated (Non-IR) and 1- to 3-week(s) old irradiation (IR)-induced senescent BJ (sBJ) cultures. The values in the middle β-Gal panel show the percentage of β-Gal positive cells. Data are mean ± SEM, *n* > 10 wells per 384-well plate from 2 independent experiment. 20× objective, Scale bar: 100 μm. (b) Western blots showing the expression of p53, p21^waf1/cip^_,_ and p16 in non-irradiated (Non-IR) BJ and 1- to 3-week(s) old sBJ cells following irradiation (IR).

**Figure S2**





**Figure S2. Co-culture and conditioned medium effect on dCK expression.** (a) Western blots showing changes in dCK expression in BJ and HCT116 isolated from co-cultures of HCT116 and normal and sBJ cells in the absence (-) or presence (+) of DAC. (b) Changes in the expression of dCK in DAC-treated HCT116 cultured in conditioned medium from BJ or sBJ cultures. The fold expression of dCK relative to β-Actin loading control is shown below the blots in (a) and (b) from one experiment.
